# Characterization of TGFβ1-induced tendon-like structure in the scaffold-free three-dimensional tendon cell culture system

**DOI:** 10.1038/s41598-024-60221-4

**Published:** 2024-04-25

**Authors:** Bon-hyeock Koo, Yeon-Ju Lee, Na Rae Park, Su Chin Heo, David M. Hudson, Aysel A. Fernandes, Chet S. Friday, Michael W. Hast, David T. Corr, Douglas R. Keene, Sara F. Tufa, Nathaniel A. Dyment, Kyu Sang Joeng

**Affiliations:** 1 McKay Orthopaedic Research Laboratory, Department of Orthopaedic Surgery, Perelman School of Medicine, University of Pennsylvania, Philadelphia, PA 19104-6081 USA; 2 Research and Development Division, BioBricks Co., Ltd, Pohang, 37673 Republic of Korea; 3 Department of Molecular Medicine, Cell and Matrix Research Institute, School of Medicine, Kyungpook National University, Daegu, 41944 Republic of Korea; 4 Department of Orthopaedics and Sports Medicine, University of Washington, Seattle, WA 98195 USA; 5 Center for Modeling, Simulation, and Imaging in Medicine (CeMSIM), Department of Biomedical Engineering, Rensselaer Polytechnic Institute, Troy, NY 12180-3590 USA; 6 Micro-Imaging Center, Shriners Children’s, Portland, OR 97239 USA

**Keywords:** Biomedical engineering, Tissue engineering

## Abstract

The biological mechanisms regulating tenocyte differentiation and morphological maturation have not been well-established, partly due to the lack of reliable in vitro systems that produce highly aligned collagenous tissues. In this study, we developed a scaffold-free, three-dimensional (3D) tendon culture system using mouse tendon cells in a differentially adherent growth channel. Transforming Growth Factor-β (TGFβ) signaling is involved in various biological processes in the tendon, regulating tendon cell fate, recruitment and maintenance of tenocytes, and matrix organization. This known function of TGFβ signaling in tendon prompted us to utilize TGFβ1 to induce tendon-like structures in 3D tendon constructs. TGFβ1 treatment promoted a tendon-like structure in the peripheral layer of the constructs characterized by increased thickness with a gradual decrease in cell density and highly aligned collagen matrix. TGFβ1 also enhanced cell proliferation, matrix production, and morphological maturation of cells in the peripheral layer compared to vehicle treatment. TGFβ1 treatment also induced early tenogenic differentiation and resulted in sufficient mechanical integrity, allowing biomechanical testing. The current study suggests that this scaffold-free 3D tendon cell culture system could be an in vitro platform to investigate underlying biological mechanisms that regulate tenogenic cell differentiation and matrix organization.

## Introduction

Tendons play a critical role in the musculoskeletal system by transmitting mechanical forces between muscle and bone. Their function requires great tensile strength provided by a highly organized collagen fiber structure consisting of multiple collagen fibrils primarily composed of type I collagen^1,2^. Despite their notable tensile strength, tendons are prone to injury. Tendon injuries present a challenging clinical problem because of the slow healing process and the inability to restore original structural stability and mechanical integrity^3–6^. Therefore, tendons are attractive targets for tissue engineering and regenerative medicine. However, the biological and mechanical mechanisms regulating cellular and matrix organization remain unclear, which impedes advancing tendon tissue engineering and regenerative medicine.

The transforming Growth Factor-β1 (TGFβ1) plays a pivotal role in various biological processes in tendon^7–16^*.* TGFβ signaling promotes tendon progenitor cell fate during early tendon development^13^. Mouse genetic studies have further indicated that TGFβ signaling is necessary to maintain tendon cell fate in neonatal tendon development and recruit tenocytes for functional neonatal tendon regeneration^11,16^. Recent transcriptomic analysis has demonstrated that altered TGFβ1 is associated with regenerative tendon healing in the MRL/MpJ mouse^8^. Studies from various tendon repair models also suggest that TGFβ1 is involved in tendon repair by regulating tendon matrix production and organization^9,10,12^. The activation of TGFβ signaling induces proliferation and inhibits apoptosis in tendon fibroblasts during the tendon healing process^17^. Given these well-established functions of TGFβ1 in tendon, we employed TGFβ1 to induce tendon-like structures in our 3D tendon constructs.

Tendon maturation results in dramatic cellular and matrix changes^18,19^. Tendon fibroblasts (i.e., tenocytes) are the primary cell type that drives tendon growth via extracellular matrix (ECM) production and organization^5,20,21^. Tenocyte differentiation is a multistep process that requires specific gene expression and unique morphological changes. Tendon progenitors are marked by the expression of *Scleraxis* (*Scx*), a basic helix-loop-helix (bHLH) transcription factor that is critical for tenocyte differentiation^22,23^. Cells at later stages of the lineage highly express type I collagen and tenomodulin^15,24,25^. Besides molecular changes, tenocytes undergo unique morphological alterations. Younger tendons consist of less elongated cells at a higher density positioned within immature ECM, but fully matured tendons have more elongated tenocytes at a lower density aligned longitudinally between organized collagen fibers^19^. Mature tendon cells cease proliferation and produce extracellular matrix, which results in matrix maturation and lower cell density in the postnatal tendon^18^. Despite the critical role of tenocytes in tendon maturation, the biological mechanisms regulating molecular and morphological changes in tenocytes are unclear, partly due to the lack of a reliable in vitro system.

Standard monolayer cell cultures have been well-established and successfully used to understand the regulatory mechanisms for cellular differentiation and homeostasis in many musculoskeletal tissues such as bone, cartilage, and muscle^26–29^. However, tendon cell cultures rely on the primary cells isolated from various tendons due to the lack of established tendon cell lines. In addition, the tenocyte phenotype is not maintained in monolayer culture, and there is variability in cells by isolation method, mouse age, and tendon type^30,31^. It is also challenging to study ECM organization and morphological maturation of tenocytes without a 3-dimensional (3D) environment. Therefore, much of our current understanding of tendon development and postnatal tendon maturation has come from the studies of murine genetic models^32^. Recent single-cell transcriptomic studies discovered tendon’s complex cellular landscape, consisting of intrinsic and extrinsic cell populations such as tendon fibroblasts, macrophages, endothelial cells, and pericytes^33–35^. Tendon maturation is also regulated by multiple factors, such as biological signaling pathways and various mechanical forces^36^. Due to this complexity, in vivo mouse models are limited in their ability to mechanistically investigate the independent biological or biomechanical factors that regulate tendon development and maturation. Therefore, developing a reliable 3D in vitro tendon culture system will be beneficial to overcome the limitations of the monolayer cell culture systems and animal models.

Recent scaffold-free and cell-based tissue engineering approaches have developed several unique in vitro tendon-like constructs. Tendon constructs have been generated from contracted or rolled-up monolayer cell culture and implanted to treat tendon injuries in small and large animal models^37–39^. Another approach utilized 3D dialysis-tube-based roller culture to produce fiber-like cell aggregates presenting tendon-like organization of cells and collagen fibers^7^. At the single-fiber scale, a micromold-based technique was used to generate single cellular fibers, wherein cellular self-assembly and fiber formation was directed by differentially adherent growth channels coated with fibronectin^40,41^. These scaffold-free approaches have an advantage for investigating the biological mechanism underlying tendon development, compared to scaffold-based techniques, because the construct is formed via the cells’ innate ability to self-assemble and produce extracellular matrix rather than the remodeling of a pre-existing scaffold structure. However, the application of many of these scaffold-free approaches has been limited to studies of embryonic tendon development due to high cell density and immature matrix organization.

In the current study, we developed a tissue-scale scaffold-free 3D tendon culture system by modifying the aforementioned published method for engineering single fibers via differentially-adherent growth channels^40,41^. We generated tendon-like constructs that display key similarities to developing tendons, including decreased cell density, increased thickness, and elongated cells between highly aligned extracellular matrix. Second Harmonic Generation (SHG) microscopy confirmed the formation of collagen fibers, and molecular analysis verified the tenogenic differentiation in the constructs. Our results suggest that the 3D tendon culture system using mouse tendon cells provides an in vitro platform to study underlying cellular and molecular mechanisms involved in tendon development and growth.

## Materials and methods

### Animals

All experiments were performed in accordance with relevant guidelines and regulations that were approved by the Institutional Animal Care and Use Committee (IACUC) and University Laboratory Animal Resources (ULAR) at the University of Pennsylvania (Philadelphia, Pennsylvania, USA). The study was carried out in compliance with the ARRIVE guidelines^42^. Mice were euthanized by carbon dioxide gas asphyxiation using IACUC-reviewed and -approved procedures. The male and female mice from mixed background lines were used to generate 3D tendon constructs.

### Monolayer culture for primary tail tendon cell

**Figure 1:**
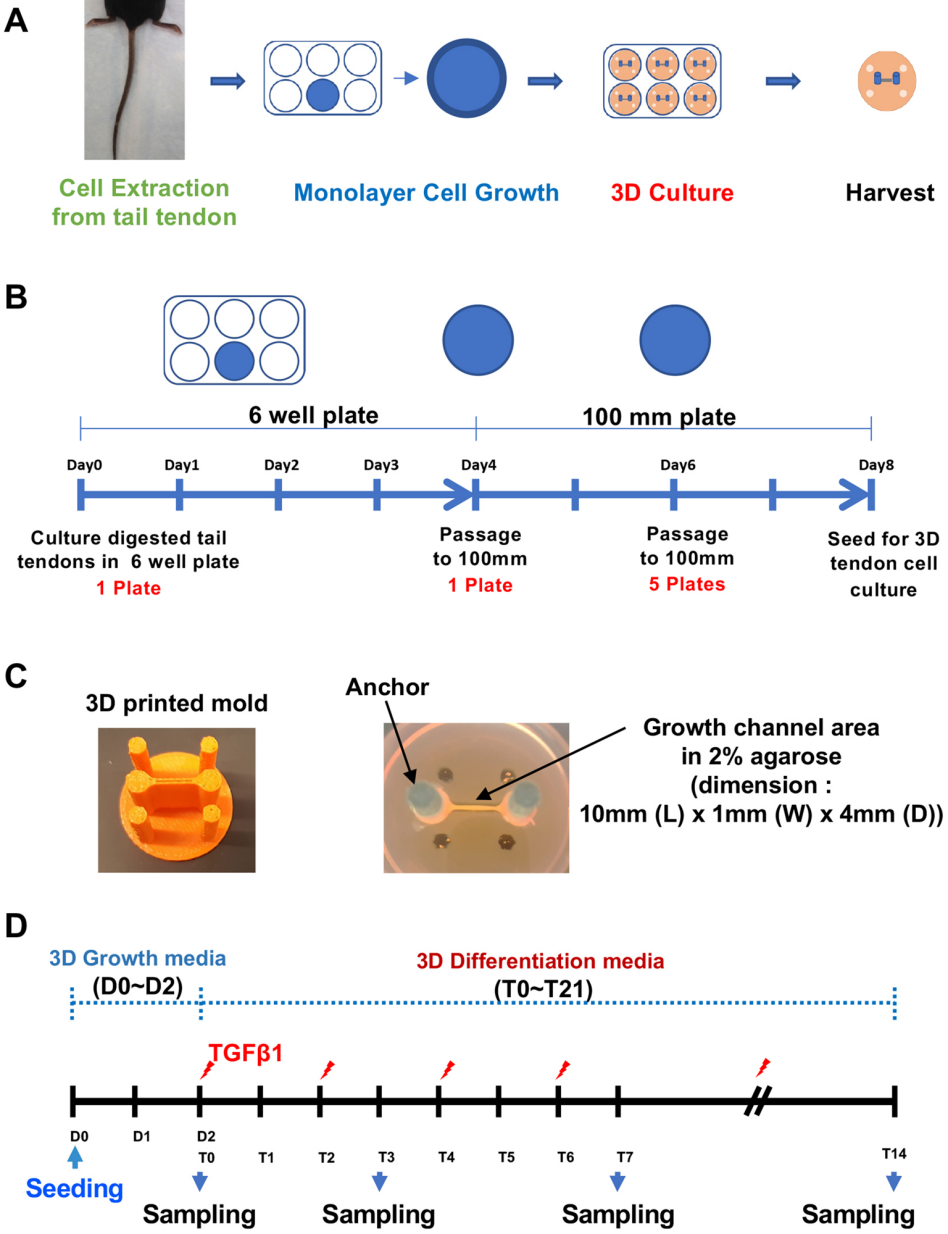
Development of 3D tendon cell culture system. (**A**) Three major steps of 3D tendon culture: cell extraction, cell growth in monolayer, and 3D culture. (**B**) The timeline of monolayer tendon cell culture after cell extraction. (**C**) The base of the 3D tendon cell culture consists of a growth channel area molded into 2% agarose using 3D-printed-mold and cylindrical anchors wrapped by hydrophilized PCL. Primary cells from the mouse tail tendon were seeded (2.5 × 10^6^ cells per construct) into the fibronectin-coated growth area to generate a 3D tendon construct. The dimension of growth area is 10 mm (length) × 1 mm (width) × 4 mm (depth). (**D**) The timeline of 3D tendon culture following monolayer culture (D, days after seeding of cells for 3D tendon culture; T, days after TGFβ1 treatment).

Tail tendons were isolated from 25 to 28 days old mice. The isolated tail tendons were digested with 5 ml of type I collagenase solution (2 mg/mL in 1× PBS, Sigma C0130) for one hour in a 37 °C incubator by gently inverting every 10 min. The digested tail tendons were cultured in six-well plates with tendon growth medium (αMEM, Gibco) supplemented with 20% Fetal Bovine Serum (FBS, New Zealand origin, Sigma F8317), 1% Penicillin–Streptomycin, and 2 mM L-Glutamine) for four days until 80–90% confluency. Then, tendon cells were passaged from a six-well plate to a 100 mm tissue culture plate and grown in tendon growth medium for two days until 80–90% confluency was reached. Finally, the tendon cells were split into five 100 mm tissue culture plates (1:5 split ratio). Cells were grown for two more days until 80–90% confluent, then harvested for 3D tendon cultures. The timeline of monolayer culture is summarized in Fig. 1 (Fig. 1A,B).

### Generation of three-dimensional (3D) tendon constructs

#### Growth channel assembly

We modified the previously published growth channel self-assembly method (32, 33). Growth channels were made using a 3D-printed mold in agarose solution (2% agarose in αMEM) on a 6-well plate (Fig. 1C). The mold was removed after gelling the agarose for 30 min to produce a growth channel of 10 mm in length, 1 mm in width, and 4 mm in depth. The agarose channels were sterilized by UV for 30 min. Aligned poly(ε-caprolactone) (PCL) nanofibrous scaffolds were prepared via electrospinning for Anchor. More specifically, a solution containing 14.3%wt/vol PCL in a 1:1 mixture of tetrahydrofuran and N, N-dimethylformamide was loaded into a 20 ml syringe. This solution was extruded at a rate of 2.5 ml/hour through an 18G stainless steel needle, with the needle charged to + 13 kV. The resulting nanofibers were collected on a rotating cylindrical mandrel with a surface velocity of 10 m/sec, ensuring the alignment of the fibers. Anchors, 3D-printed cylinders wrapped with the hydrophilized electrospun PCL, were inserted into both ends of the growth channel area (Fig. 1C), and UV sterilization was performed again for 30 min. The growth channels were then coated with Human plasma-derived fibronectin (0.375 mg/ml in 1X PBS, Corning) and dried for 20 min.

#### Three-dimensional (3D) tendon cell culture

Cells were seeded (2.5 × 10^6^ cells) into the fibronectin-coated agarose growth channel area in each well. Growth media (a-MEM supplemented with 20% fetal bovine serum, 1% penicillin–streptomycin, 2 mM L-Glutamine, and 50ug/ml L-Ascorbic acid) was added to the culture plate 10 min after seeding, and the seeded culture plates were placed into a standard cell culture incubator (37 °C, 5% CO_2_, and 95% relative humidity). Two days after seeding, growth media was replaced with differentiation media (a-MEM, supplemented with 10% fetal bovine serum, 1% penicillin–streptomycin, 2 mM L-Glutamine, and 50ug/ml L-Ascorbic acid) with vehicle (4 mM HCL containing 1 mg/ml bovine serum albumin) or TGFβ1 (5 ng/ml in 4 mM HCL containing 1 mg/ml bovine serum albumin, R&D system, 240-B-002). The differentiation media was replaced every other day for the duration of time in culture. 3D tendon constructs were incubated with BrdU (5 μg/ml) for two hours before harvest. The timeline of 3D tendon culture is summarized in Fig. 1D.

### RT-PCR and quantitative real-time PCR (qRT-PCR)

RNA was extracted with Trizol and Direct-zol RNA miniprep kits (Zymo Research, R2050) from 3D tendon constructs. cDNA was synthesized from 500 ng of total RNA using iScript Reverse Transcription (Bio-Rad, 1708841). The qRT-PCR was performed using SYBR green master mix (Pec, 4385612) on Quantstudio 6. The sequences of primers are listed in Table 1.

**Table 1:** Primer sequences for qRT-PCR.

Gene	Forward (5'–3')	Reverse (5'–3')
mHprt	AGCGATGATGAACCAGGTTA	GTTGAGAGATCATCTCCACC
mScx	AAGACGGCGATTCGAAGTTAGAAG	TCTCTCTGTTCATAGGCCCTGCTCATAG
mTnmd	CTTTACTAGGCTACTACCCATACCCCTACT	ATATATTGGCTAACAGAAGGTTAAGCGTTT
mCol1a1	TTGGGGCAAGACAGTCATCGAAT	TTGGGGTGGAGGGAGTTTACACGAA

### Histological analyses

3D tendon constructs were harvested at various stages and fixed in 4% paraformaldehyde solution for one hour at 4 °C. The fixed samples were paraffin-embedded and sectioned with 6 µm thickness using a microtome. These sections were used for Hematoxylin and Eosin (H&E) staining, BrdU staining (Abcam, ab125306), DAPI staining, and TUNEL assay (Abcam, ab206386). Nucleus shape and cell density in the peripheral layer of the 3D tendon structure were analyzed using DAPI-stained samples. The shape of the nucleus was characterized by the ratio of the nuclear transverse length to its length in the direction of the channel length.

### Second harmonic generation

The paraffin-embedded sections were mounted with an anti-fade DAPI solution (Invitrogen, P36935) for imaging. Second Harmonic Generation (SHG) Images were generated by a Leica TCS SP8 MP multiphoton microscope (Leica Microsystems, Buffalo Grove, IL) equipped with a Coherent Chameleon Vision II Ti–Sapphire laser (Coherent, Santa Clara, CA). The images were acquired using excitation at 880 nm. DAPI images were acquired using 405 nm excitation and detection from 414 to 471 nm. The objective lens was a 40 ×/1.3 NA HC PL APO CS2 oil immersion lens. Z-stacks were acquired using a zoom of 2, a scan speed of 600 Hz, bidirectional scanning, and 6-line averaging. The lateral (x–y) pixel size was 0.0865 microns; the z-step size was 0.50 microns. The intensity of the SHG signal was assessed using ZEISS ZEN software (Carl Zeiss Microscopy, ZEN 3.9, Jena, Germany). Relative arbitrary values for images were calculated based on the SHG fluorescence area. Collagen alignment was determined by quantifying the angle of collagen fibers to the horizontal line, utilizing ImageJ software (Fiji 2.15.0). Initially, SHG images were converted to 64-bit in Image J, and the region of interest was selected in the peripheral layer of tendon constructs. Collagen alignment angles were measured relative to the horizontal axis, parallel to the outer boundary of the peripheral layer. Lines indicating collagen direction were drawn following SHG signals representing collagen fibers. To ensure unbiased selection, all collagen fibers for quantification were counted in a single-blinded manner.

### Electron microscopy analysis for collagen fibril

The collagen fibril analysis was previously described^43^. Briefly, 3D tendon constructs were harvested and fixed in the fresh buffer (1.5% glutaraldehyde/1.5% paraformaldehyde (Tousimis) with 0.05% tannic acid (Sigma) in DPBS) at 4 °C overnight. The isolated 3D tendon constructs were post-fixed in 1% OsO4 and rinsed in DMEM. The samples were then gradually dehydrated in a series of ethanol to 100%. Finally, samples were rinsed in propylene oxide, infiltrated in Spurrs epoxy, and polymerized at 70 °C overnight. A FEI G20 TEM was used for transmission electron microscopy (TEM) images which visualize transverse sections of collagen fibrils at multiple magnifications. Collagen fibril diameter was measured using ImageJ software (Fiji 2.15.0). TEM images of six constructs per group were analyzed. Total of 3452 fibrils in vehicle-treated constructs and 3507 fibrils in TGFβ1-treated constructs were counted.

#### Pyridinoline cross-linking analysis

The collagen pyridinoline cross-link content was measured using fluorescence monitoring with reverse-phase HPLC. Briefly, pyridinoline cross-links were analyzed in samples by HPLC after acid hydrolysis with 6 M HCl for 24 h at 108 °C. Dried samples were dissolved in 1% (v/v) n-heptafluorobutyric acid for quantitation of hydroxyproline (HP) by reverse-phase HPLC and fluorescence monitoring as previously described^44^. Collagen content was determined using hydroxyproline assays as previously described^44^. Total collagen was calculated using a ratio of 300 hydroxyproline residues per triple helix. Percent collagen is based on the lyophilized dry weight of tissue.

### Uniaxial biomechanical testing

Uniaxial testing was performed similar to previously established protocols^45–47^. All specimens were kept frozen at -20 °C until the day of testing. After thawing, a custom laser device was used to measure the cross-sectional area (mm^2^). The ends of each specimen were placed between two sandpaper tabs and held together with cyanoacrylate glue to prevent specimen slippage during the mechanical test. Samples were gripped with custom aluminum fixtures and mounted onto a universal test frame (Instron 5542, Instron Inc., Norwood, MA) equipped with a 10N load cell. All testing was conducted in a phosphate-buffered saline bath maintained at room temperature (23 °C). During the tests, each sample was preloaded to 0.02 N, followed by ten cycles of preconditioning between 0.02 and 0.04 N. The specimens then underwent a quasi-static ramp to failure at a displacement rate of 0.01 mm/s. Force, displacement, and time data were collected at 100 Hz. Force–displacement curves were analyzed to quantify tissue stiffness (N/mm, defined as the slope of the linear region) and ultimate force. Force and displacement data were normalized by specimen cross-sectional area (CSA) and gauge length, respectively, to convert to stress and strain. The resulting stress–strain curves were assessed to determine elastic modulus (MPa, defined as the slope of the linear region) and failure stress (MPa). Work to failure (mJ) and toughness (mJ/m^3^) were determined by calculating the areas under the force–displacement and stress–strain curves, respectively.

### Statistical analysis

Results are expressed as mean ± SD. At least three 3D tendon constructs per group were analyzed for each experiment. Differences between values were analyzed by two-way ANOVA. *p* < 0.05 is considered significant.

## Results

### TGFβ1 treatment induced tendon-like tissue in 3D tendon constructs

**Figure 2:**
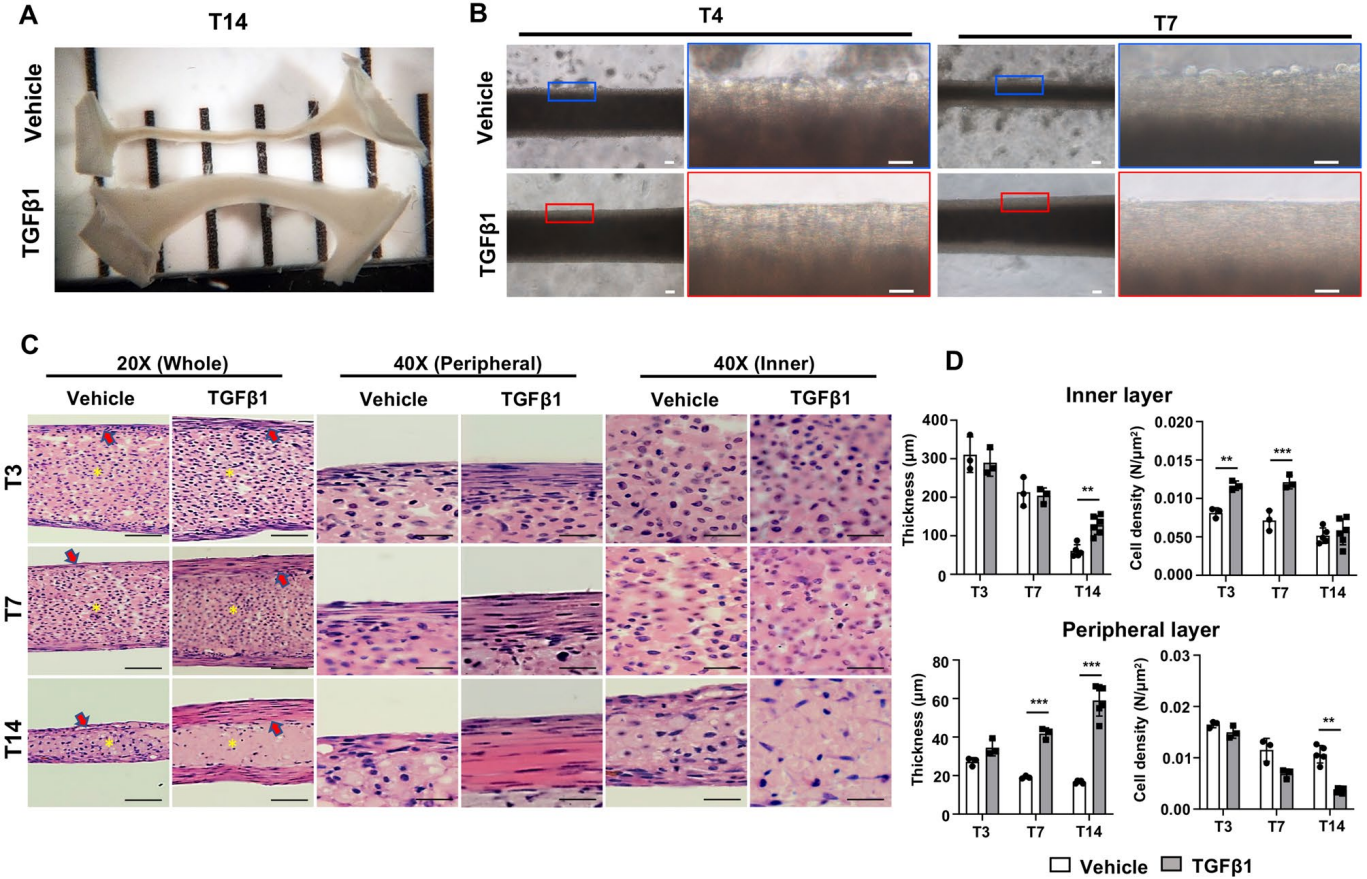
Histological analyses of the 3D tendon constructs. (**A**) Dissecting microscope images of 3D tendon constructs at 14 days after TGFβ1 or vehicle treatment (T14). (**B**) Higher magnification images of 3D tendon constructs at T4 and T7 after TGFβ1 or vehicle treatment. Scale bars in lower magnification images indicate 100 μm, and scale bars in higher magnification images indicate 50 μm. (**C**) H&E-stained longitudinal sections from vehicle- and TGFβ1-treated 3D tendon constructs at each stage (T3, T7, and T14). Scale bars in lower magnification images indicate 100 μm, and scale bars in higher magnification images indicate 50 μm. Yellow asterisks indicate inner layers, and red arrows indicate peripheral layers. (**D**) Quantification results of thickness and cell density of inner and peripheral layer (** indicates P < 0.01 and *** indicates P < 0.001, n = 3 ~ 5 constructs per group).

The seeded cells in the fibronectin-coated channel self-assembled into a contiguous, uniaxial construct within 24 h after the seeding. However, the thickness of the constructs was not maintained with regular growth media (Fig. 2A, vehicle). Microscopic analysis at higher magnification showed many cells budding out from the surface of the vehicle-treated constructs at T4 and T7 (Fig. 2B, upper panels). To analyze the structure of the vehicle-treated 3D constructs, we collected the constructs at different time points and performed histological analysis using H&E-stained longitudinal sections (Fig. 2C). Lower magnification images confirmed that the whole thickness of the vehicle-treated construct was reduced at T14 (Fig. 2C, 20 × whole, left panels). We also found that the 3D tendon constructs contained two cell layers, inner (Fig. 2C, yellow asterisks) and peripheral (Fig. 2C, red arrows) layers. Dramatic decrease in inner layer thickness was observed in vehicle-treated constructs at T14 (Fig. 2C, 20 × whole, left panels). The quantification results verified the gradual decrease in the inner layer thickness of vehicle-treated constructs (Fig. 2D, upper left panel, white bar). There was no significant change in cell density of the inner layer in vehicle-treated constructs (Fig. 2D, upper right panel, white bar, *p* = 0.067 (T3 vs. T14)). Lower magnification images showed a slight reduction in peripheral layer thickness of vehicle-treated constructs (Fig. 2C, 20 × whole, vehicle), which is confirmed by the higher magnification image (Fig. 2C, 40 × peripheral, vehicle). In our quantification analysis, we verified slight reduction in peripheral layer thickness in vehicle-treated constructs (Fig. 2D, bottom left panel, white bars, *p* < 0.001 (T3 vs. T14)). A gradual reduction in cell density of the peripheral layer was observed in vehicle-treated constructs ((Fig. 2D, bottom right panel, white bars*, p* = 0.002 (T3 vs. T14)). Previous studies suggested that additional growth factors could be essential for maintaining the engineered tendon constructs^7,38,40^. Transforming growth factor-beta (TGFβ) is a well-known growth factor and critical for tendon development, maturation, healing, and tenogenic differentiation in vivo and in vitro^7–16^. These prior findings prompted us to use TGFβ1 in our 3D tendon culture. Interestingly, the thickness of the construct was relatively well-preserved with TGFβ1 treatment compared to that of the vehicle-treated construct at T14 (Fig. 2A). Microscopic analysis at higher magnification further confirmed that TGFβ1 treatment preserved the thickness of constructs and maintained the smooth surface without the budding cells at earlier stages, such as T4 and T7 (Fig. 2B, bottom panels). Histological analysis verified that the thickness of the TGFβ1-treated construct was relatively preserved when compared to vehicle-treated constructs (Fig. 2C, 20 × whole, right panels). The inner layer thickness of TGFβ1-treated constructs was gradually reduced (Fig. 2C, 20 × whole) at T14, which is confirmed by quantification (Fig. 2D, upper left panel, gray bars, *p* < 0.001 (T3 vs. T14)). However, the inner layer of TGFβ1-treated construct was significantly thicker than that of vehicle-treated constructs at T14 (Fig. 2D, upper left panel, *p* = 0.004). TGFβ1-treated constructs displayed higher cell density in the inner layer at T3 and T7 when compared to vehicle-treated constructs, but there was no difference at T14 (Fig. 2D, upper right panel, *p* = 0.007 (T3), *p* < 0.001 (T7), *p* = 0.867 (T14)). The peripheral layer of TGFβ1-treated constructs became markedly thicker at T14 when compared to early stages such as T3 and T7 (Fig. 2C, 20 × whole and 40 × peripheral, TGFβ1). The quantification results revealed dramatically increased peripheral layer thickness of TGFβ1-treated constructs at T14 (Fig. 2D, bottom left panel, gray bars, *p* = 0.002 (T3 vs. T14)). Interestingly, the higher magnification image shows that the peripheral layer of the TGFβ1-treated constructs presented a tendon-like tissue formation, including elongated cells between a highly aligned extracellular matrix and decreased cell density (Fig. 2C, 40 × peripheral, TGFβ1). Our quantification results verified the dramatic reduction in the peripheral layer cell density of TGFβ1-treated constructs throughout the stages (Fig. 2D, bottom right panel, gray bars, *p* < 0.001 (T3 vs. T7), *p* < 0.001 (T3 vs. T14)). Overall, these findings strongly suggest that TGFβ1 is required for promoting proper aggregation and self-assembly of the 3D tendon constructs, and TGFβ1 treatment induces tendon-like tissue formation in the peripheral layer of the constructs.

### TGFβ1 treatment increased cell proliferation in 3D tendon constructs

**Figure 3:**
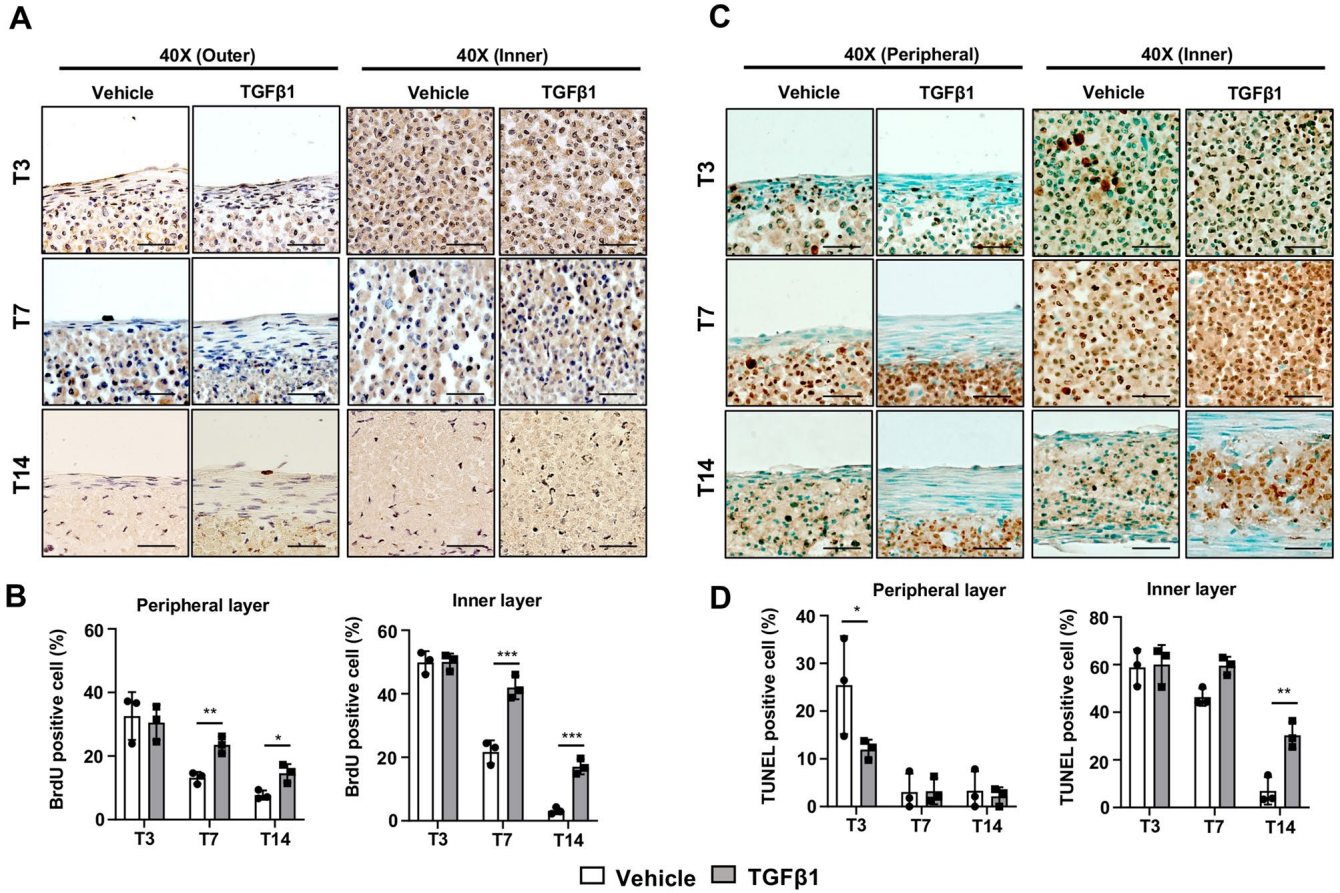
Cellular proliferation and apoptosis in the 3D tendon constructs. (**A**) BrdU staining of peripheral and inner layers in vehicle- and TGFβ1-treated 3D tendon constructs at each stage (T3, T7, and T14). Scale bars indicate 50 μm. (**B**) Quantification results of BrdU-positive cells (brown) in peripheral and inner layers of 3D tendon constructs (** indicates P < 0.01 and *** indicates P < 0.001, n = 3). (**C**) TUNEL assay of peripheral and inner layers in vehicle- and TGFβ1-treated 3D tendon constructs at each stage (T3, T7, and T14). Scale bars indicate 50 μm. (D) Quantification results of TUNEL-positive cells (brown) in peripheral and inner layers of 3D tendon constructs (* indicates P < 0.05 and ** indicates P < 0.01, n = 3 constructs per group).

The decreased cell density in the peripheral layer of 3D tendon constructs prompted us to investigate cellular proliferation and apoptosis. To study cellular proliferation, we performed BrdU staining with longitudinal sections of 3D tendon constructs (Fig. 3A). The quantification results show that both vehicle- and TGFβ1-treated constructs exhibit gradual reduction in cellular proliferation in both layers with time in culture (Fig. 3B). However, TGFβ1-treated constructs display significantly higher proliferation rates at T7 (*p* = 0.005 (peripheral), *p* < 0.001 (inner)) and T14 (*p* = 0.022 (peripheral), *p* < 0.001 (inner)). To examine apoptosis, we performed the TUNEL assay using longitudinal sections of 3D tendon constructs (Fig. 3C). In the peripheral layer, vehicle-treated constructs display a higher apoptosis rate than TGFβ1-treated constructs at T3 (*p* = 0.019), but no difference was observed at T7 (*p* = 0.999) and T14 (*p* = 0.989) (Fig. 3D, left panel). In the inner layer, both constructs present high apoptosis rates at T3 and T7, but the apoptosis rate is dramatically reduced at T14 (Fig. 3D, right panel). Interestingly, TGFβ1-treated constructs show a significantly higher apoptosis rate at T14 when compared to vehicle-treated constructs in inner layer (*p* = 0.001). Overall, TGFβ1 treatment relatively increased cellular proliferation in both peripheral and inner layers at later stages (T7 and T14) but only prevented cellular apoptosis in the peripheral layer at early stage (T3) and enhanced apoptosis in inner layer at late stage (T14).

### TGFβ1 stimulated the production of a dense, aligned collagen matrix

**Figure 4:**
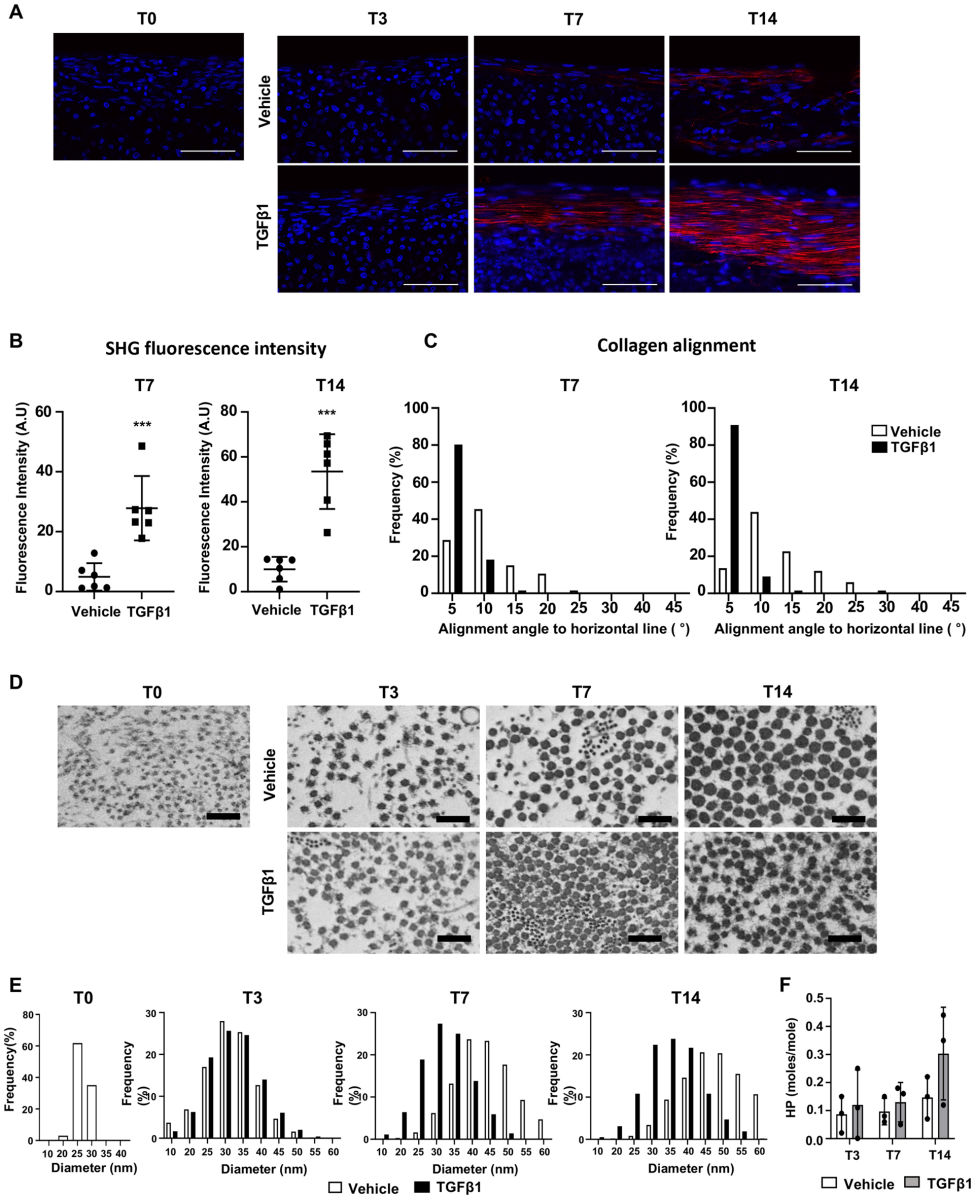
Collagen fiber formation, modification and fibrillogenesis in the 3D tendon constructs. (**A**) Second harmonic generation (SHG) microscopy images (collagen fiber formation) of peripheral layers of vehicle- and TGFβ1-treated 3D tendon constructs at each stage (T0, T3, T7, and T14). Scale bars indicate 50 μm. (**B**) Quantification of SHG signal intensity at T7 and T14 (*** indicates P < 0.001, n = 6 constructs per group). (**C**) Quantification of collagen fiber alignment using SHG images at T7 and T14 (n = 6 constructs per group at each stage). (**D**) Transmission electron microscopy (TEM) microscopy images of peripheral layers of vehicle- and TGFβ1-treated 3D tendon constructs at each stage (T0, T3, T7, and T14). Scale bars indicate 500 nm. (**E**) Quantification of collagen fibril diameter using TEM images from vehicle- and TGFβ1-treated 3D tendon constructs at each stage (n = 3 constructs per group at each stage). (**F**) hydroxylysylpyridinoline level of vehicle- and TGFβ1-treated 3D tendon constructs at each stage (n = 3 constructs per group at each stage).

The highly organized collagen fiber is a major feature of tendon structure. Tendon-like tissue formation in the peripheral layer of the TGFβ1-treated constructs prompted us to assess collagen fiber formation using Second Harmonic Generation (SHG) microscopy (Fig. 4A). A gradual increase in SHG signal was observed in peripheral layers of both vehicle and TGFβ1-treated constructs, but TGFβ1-treated constructs exhibited stronger SHG signal. Specifically, no SHG signal was detected in the peripheral layer of vehicle-treated constructs at T3, and only a weak signal was detected at T7 and T14. SHG signal was also undetectable in the TGFβ1-treated constructs at T3, but strong linear SHG signals were detected at T7 and T14. The quantification results revealed that TGFβ1-treated constructs exhibited significantly higher SHG signal intensity at T7 and T14 (*p* < 0.001 for both T7 and T14). No SHG signals were detected in the inner layers of either construct at all stages. To assess the organization of collagen fibers, we examined their alignment in the 3D tendon construct using Second Harmonic Generation (SHG) images (Fig. 4C). Collagen alignment was measured by the angles relative to the horizontal axis, parallel to the peripheral layer's outer boundary. In this measurement, a degree of 0 would indicate perfect alignment, with greater angles indicating less aligned collagen fibers. Most collagen fibers in TGFβ1-treated constructs exhibited alignment angles under 10 degrees, with the peak distribution between 0° and 5° (Fig. 4C, black bar). Collagen fibers in vehicle-treated constructs displayed alignment angles ranging from 5° to 25°, with a peak distribution between 5° and 10° (Fig. 4C, white bar). These results suggest that TGFβ1-treated constructs contain more aligned collagen fibers than vehicle-treated constructs. Overall, our SHG image analysis suggests that TGFβ1 treatment induced the production of a denser, more aligned collagen matrix in the peripheral layer of the 3D tendon construct.

We further examined collagen fibril ultrastructure and cross-linking. Transmission electron microscopy (TEM) was performed using 3D tendon constructs to measure collagen fibril diameter. TEM images visualized transverse sections of collagen fibrils, and both constructs showed gradual growth of collagen fibrils diameter throughout the stages (Fig. 4D). The quantification results demonstrated the gradual increase in collagen fibril diameter in vehicle-treated constructs (Fig. 4E, white bars). Specifically, at T0, the constructs displayed a narrow range of collagen fibril sizes, with the greatest proportion of collagen fibrils at a diameter of 25 nm. The size (diameter) of the greatest proportion of fibrils gradually increased to 30 at T3, 40 at T7, and 45 at T14. TGFβ1-treated constructs exhibited a similar growth of collagen fibril diameter at T3 when compared to vehicle-treated constructs (Fig. 4E, black bar). Interestingly, TGFβ1 treatment yielded smaller diameter fibrils compared to vehicle controls, indicated by the greatest proportion of collagen fibril at a diameter of 30 for T7 and 35 for T14. Collagen cross-linking analyses were performed to assess the status of collagen modification. The cross-linking analysis revealed no significant difference in hydroxylysylpyridinoline between vehicle- and TGFβ1-treated tendon constructs at all stages (*p* = 0.969 (T3), *p* = 0.970 (T7), *p* = 0.218 (T14)). It should be noted that while hydroxylysylpyridinoline provides a useful measure of stable (irreversible) cross-links, it is not a measure of the total cross-links. Overall, our collagen analyses indicate that the peripheral layer of the 3D tendon construct undergoes tendon-like tissue formation, and TGFβ1 enhances collagen fiber formation and organization but inhibits the lateral growth of collagen fibril.

### TGFβ1 induced morphological maturation and tenogenic differentiation

**Figure 5:**
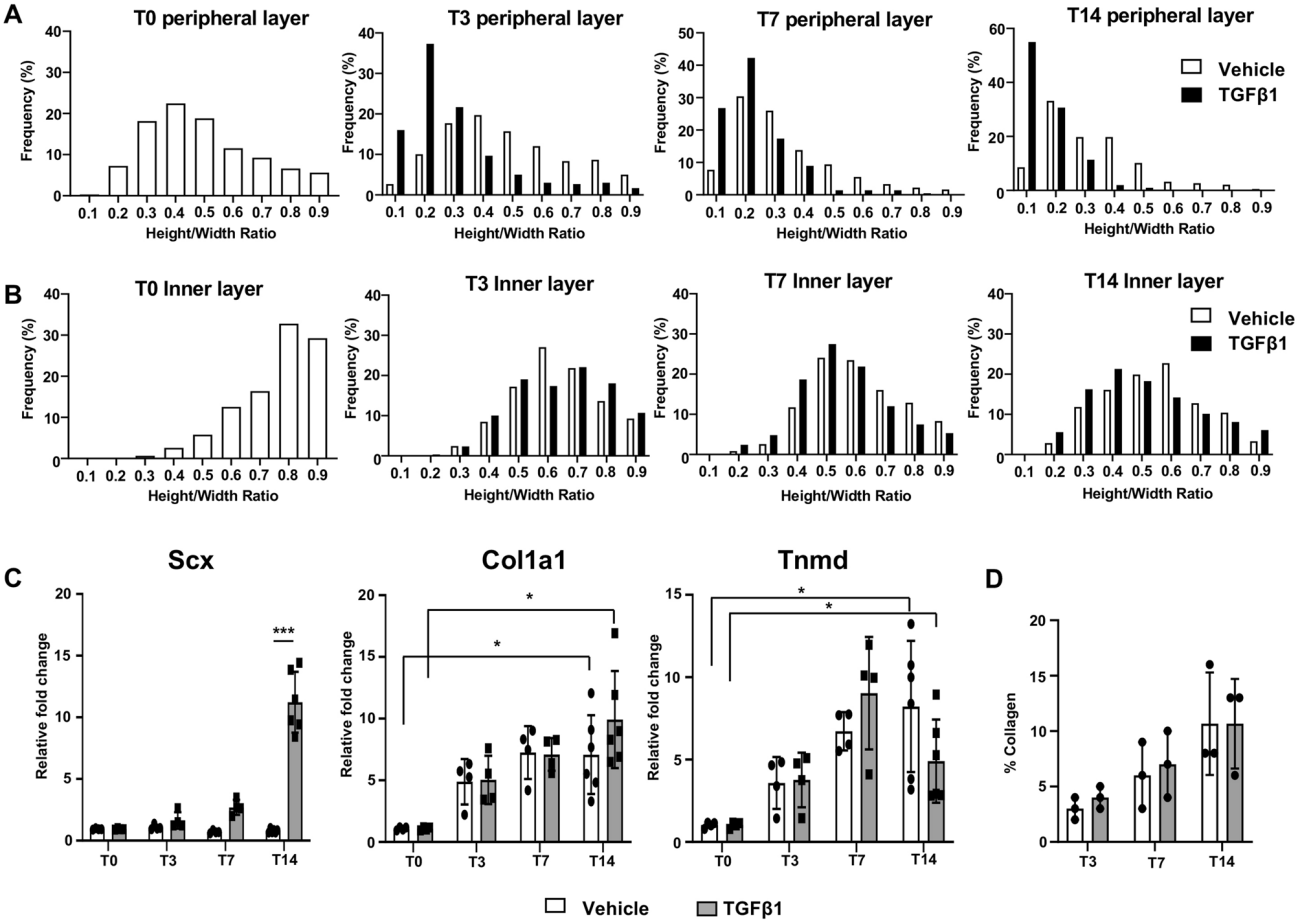
Morphological and molecular changes of cells in the 3D tendon constructs. The ratio of the nuclear transverse length to its length in the direction of the channel length of cells in peripheral (**A**) and inner (**B**) layers (values closer to 1.0 indicates a rounder cell shape, and values closer to 0 indicate a more elongated cell shape). n = 3 constructs per group. (**C**) Quantitative real-time PCR (qRT-PCR) results for tenogenic gene expression in 3D tendon constructs, including Scleraxis (Scx), Tenomodulin (Tnmd), and type 1 collagen (Col1a1). * indicates P < 0.05 and *** indicates P < 0.001 (n = 4–6 constructs per group). (**D**) Relative collagen amount of vehicle- and TGFβ1-treated 3D tendon constructs at each stage (n = 3 constructs per group at each stage).

To investigate cellular phenotypes in 3D tendon constructs, we first examined the morphological changes of cells in the 3D tendon construct (Fig. 5A,B). We measured the ratio of the nuclear transverse length to its length in the direction of the channel length. In this measurement, a value of 1.0 would indicate a perfect circle, and 0 would be a line in the direction of the channel length. Therefore, nuclear height/width ratios closer to 1.0 indicates a rounder cell shape, whereas values less than 1 indicate a cell shape more elongated in the direction of construct length, with smaller values indicating greater cellular elongation. Gradual cell elongation was observed in the peripheral layer of both vehicle- and TGFβ1-treated constructs, with the TGFβ1-treated constructs exhibiting more elongated cells than vehicle-treated constructs throughout the stages (Fig. 5A). Specifically, the cells in the 3D tendon constructs were relatively oval shaped at T0, which is evident with the major distributed values between 0.3 and 0.5 (Fig. 5A, T0 peripheral layer). Vehicle-treated constructs exhibited minimal morphological changes at T3, and the cells became elongated at T7 and T14, which is evident with the major distributed values between 0.2 and 0.3 (Fig. 5A, white bar). The cells in TGFβ1-treated constructs presented dramatic morphological changes at T3 and T7, which is evident with most of the values distributed between 0.1 and 0.3 (Fig. 5A, black bar). The cells became further elongated at T14, with the greatest proportion of cells at a ratio of 0.1. Within the inner layer, the cells only show morphological changes between T0 and T3, but no further changes were observed (Fig. 5B). Additionally, TGFβ1 treatment does not appear to affect the morphological maturation of the inner layer cells (Fig. 5B). These data suggest that the cells in the peripheral layer undergo morphological maturation, and TGFβ1 can be used to enhance this morphological maturation.

To assess tenogenic differentiation, we examined the expression of three different tenogenic gene markers, *Scleraxis (Scx)*, *Tenomodulin (Tnmd)*, and Collagen type 1 (*Col1a1*) (Fig. 5C). First, TGFβ1 treatment significantly induced the expression of *Scleraxis*, a critical transcription factor for tenogenic differentiation (Fig. 5C, Scx). TGFβ1 treatment increased the expression of *Scleraxis* at T14 by tenfold compared to T0 (*p* < 0.001), while Scleraxis expression was not changed at all stages of vehicle-treated constructs (*p* = 0.09 (T0 vs. T14)). Collagen type 1 and *Tenomodulin*, relatively later tenogenic markers, were significantly increased in later stages in both vehicle- and TGFβ1-treated constructs (Col1a1: *p* = 0.006 (T0 vs. T14 for vehicle-treated), *p* = 0.002 (T0 vs. T14 for TGFβ1-treated), Tnmd: *p* = 0.008 (T0 vs. T14 for vehicle-treated), *p* = 0.017 (T0 vs. T14 for TGFβ1-treated)), but no significant changes were observed between the two treatments (Fig. 5C, Col1a1 and Tnmd, *p* > 0.5 at all stages). Consistent with the gene expression analysis, relative collagen amount is similar between vehicle- and TGFβ1-treated constructs (Fig. 5D). Overall, gene expression data suggest that TGFβ1 may promote tenogenic cell differentiation in the 3D tendon constructs.

### TGFβ1 treatment enhanced the mechanical properties of 3D tendon constructs

To measure the mechanical properties of 3D tendon constructs, we performed uniaxial biomechanical testing with samples isolated at T14. Vehicle-treated tendon constructs were extremely delicate, so they did not possess sufficient mechanical integrity to allow for biomechanical characterization and broke prior to the completion of mechanical testing. On the other hand, TGFβ1-treated samples were strong enough to perform mechanical testing. The measured mechanical property values are summarized in Table 2. These mechanical testing data suggest that TGFβ1 treatment enhanced the mechanical properties of 3D tendon constructs.

**Table 2:** Mechanical properties of TGFβ1-treated 3D tendon constructs at T14 (n = 7, value ± standard deviation).

Cross-sectional area (mm^2^)	0.17 ± 0.10
Max load (N)	0.43 ± 0.14
Stiffness (N/mm)	0.23 ± 0.07
Max stress (MPa)	3.52 ± 2.43
Modulus (MPa)	20.55 ± 9.92
Energy to failure (mJ)	0.50 ± 0.26
Max strain (mm/mm)	0.19 ± 0.06
Toughness (mJ/m^3^)	0.39 ± 0.38

## Discussion

In this study, we successfully generated in vitro 3D constructs using mouse tail tendon cells. Our culture system confers distinct advantages over the original method involving the engineering of single fibers via differentially-adherent growth channels^40,41^. We demonstrated that our culture generated a relatively larger size of constructs. This increased size facilitates easy handling, enables straightforward mechanical analysis, and permits a comprehensive range of histological and molecular analyses. Utilizing mouse tendon cells also provides a potential for biologic investigation using genetic manipulation upon the mouse genetic background. These constructs will be a useful tool to investigate cellular and molecular mechanisms regulating tendon development in vitro*.* They will also contribute to the understanding of scaffold-free bioengineered graft models.

Previous studies suggested the potential requirement of growth factors for the formation of engineered tendon constructs^7,38,40^. Our results also indicated that TGFβ1 enhances the formation of 3D tendon constructs. The concentration (5 ng/ml) of TGFβ1 was determined after preliminary testing of three different dosages, including 3 ng/ml, 5 ng/ml, and 10 ng/ml. Specifically, the constructs with 3 ng/ml of TGFβ1 still contains budding cells from the surface, and the constructs with 5 ng/ml and 10 ng/ml of TGFβ1 had no difference in the gross morphology of the constructs. We also compared the TGFβ1 and TGFβ3 at 5 ng/ml concentration as a preliminary study. TGFβ3-treated constructs with 5 ng/ml still showed budding cells on the surface of the 3D tendon constructs, which suggests that TGFβ1 is more efficient, respectively. Therefore, we only utilized TGFβ1 for the current studies. Besides TGFβ, the functions of several growth factors, such as BMPs, Connective tissue growth factor (CTGF), and fibroblast growth factors (FGFs), have been investigated in physiological and pathological tendon conditions using in vivo and in vitro models^15,48–51^. Upon the existence of recombinant proteins of these growth factors, our 3D tendon constructs can be used to test the function of these growth factors in tenogenic cell differentiation and matrix formation.

TGFβ1 treatment induced tendon-like structure in the peripheral layer of constructs, exhibiting increased thickness with decreased cell density, increased matrix production, and elongated cells between highly aligned extracellular matrix. We also characterized collagen fibrillogenesis, tenogenic differentiation, and biomechanical properties of the 3D tendon constructs. The decreased cell density with increased thickness in the peripheral layer suggests that TGFβ1 treatment induces the production of extracellular matrix. TGFβ1 dramatically induced the expression of *Scleraxis*, the critical transcription factor for tenogenic differentiation. However, the expression of *Col1a1* and *Tenomodulin* were not changed by TGFβ1 treatment when compared to vehicle-treated constructs. These data suggest that cells undergo early tenogenic differentiation without further molecular maturation with TGFβ1 treatment. We carefully speculate that the early arrested cell fate could explain the smaller collagen fibril diameters and increased cell proliferation in TGFβ1-treated tendon constructs when compared to control constructs.

The success of the initial monolayer culture is critical for generating 3D tendon constructs. In our preliminary study, the tail tendon cells obtained from the mice older than one month grew too slowly in monolayer culture, so we could not obtain enough cells for 3D tendon culture in the proper timeline. However, tail tendons from younger mice, e.g., 25–28 days old, were able to produce sufficient cell numbers for the studies herein. We assume that this could be attributed to tendons from young mice having more progenitor cells, which can proliferate in in vitro monolayer cell culture. Therefore, we only isolated tail tendon cells from mice younger than one month old.

We only used mouse tail tendon cells for the current study because we could isolate enough cells for 3D tendon culture from tail tendon consistently. We successfully generated six 3D tendon constructs from a single mouse tail by rigorously adhering to the timelines outlined in the Method section for monolayer cell culture. In our experience, attempting to derive enough cells from either the Achilles or Patellar tendons of a single mouse, even during the monolayer cell culture stage, was never successful. In a preliminary investigation, we combined ten Achilles or patellar tendons from five mice for 3D tendon cultures; however, the cell numbers during the monolayer culture were insufficient to meet the specified timeline. Despite this, we are optimistic that aggregating bulk tendons (exceeding ten) from numerous mice or developing tailored protocols for Achilles and patellar tendons would yield an ample supply of cells for successful 3D tendon culture. One of the future research directions using this platform is to compare the 3D tendon constructs generated using cells from different tendons (e.g., flexor, extensor) to identify whether the tissue origin of cells influences the development, structure, and function of engineered 3D tendon constructs^52^. For future translational applications of our culture system, we are also planning to use bovine or human tendon cells to generate 3D tenon constructs.

In our 3D tendon culture systems, the peripheral and inner layers of the constructs exhibited a different tissue structure. We speculate that this is due to the nutritional deficiency and lack of static tension in the center region. This phenomenon was also described in a previous culture study^7^. The potential solution for this issue could be the addition of mechanical stimulation to increase diffusion of nutrients into the construct. Additionally, our prior scaffold-free work at the single-fiber scale, which is not subject to such diffusion-based nutritional deficiency, has demonstrated that cyclic tensile loading can be utilized to improve the tensile strength as well as elastic modulus and to increase nuclear elongation of engineered tendon fibers^41^. Hence, we are currently developing a bioreactor in which we can apply cyclic loading to the 3D tendon constructs. Investigating the combinatory effects of the cyclic loading and growth factors on the formation of 3D tendon constructs will be an interesting future direction.

Our mechanical testing data suggest that TGFβ1 treatment enhanced the mechanical properties of 3D tendon constructs, but the constructs are not as mechanically mature as mouse Achilles tendons. The average cross-sectional area of TGFβ1-treated constructs (0.17 mm^2^) was similar to that of the previously reported mouse Achilles tendons at four weeks of age (0.17 mm^2^)^43,53^. But other mechanical properties of the TGFβ1-treated constructs were much lower than those of the mouse Achilles tendons at four weeks of age^43,53^. Specifically, the average stiffness and modulus of TGFβ1-treated constructs are 0.23 N/mm and 20.55 MPa, but the average stiffness and modulus of mouse Achilles tendons at four weeks of age are 50.35 N/mm and 544 MPa. These immature mechanical properties of 3D tendon constructs can be partly explained by immature collagen fibrillogenesis, showing the diameter range is between 0 and 60 nm. The mature mouse Achilles tendon exhibits that the distribution of collagen fibril diameter ranges from 20 to 200 nm^53^. Another potential factor contributing to the immature mechanical properties of 3D tendon constructs is the composition and the expression timing of different collagens. Type 1 collagen is the most abundant matrix protein in tendon^54^, but both type 1 and type 3 collagens are expressed in tendon^55^. Type 1 collagen provides tensile strength as a stiff fibrillar protein, while type 3 collagen increases elasticity by forming an elastic network^56^. Hence, the ratio of type 1 to type 3 collagen influences the biomechanical properties of the tendon. Throughout the tendon healing process, the initial deposition and organization of type 3 collagen play a crucial role in the subsequent type 1 collagen-based matrix remodeling^57^. We speculate that the unique ratio and expression timing of type 1 and type 3 collagens in 3D tendon constructs potentially resulted in immature biomechanical properties. Investigating the intricate dynamics of matrix proteins and its impact on mechanical properties will be a captivating future research with our 3D tendon constructs.

One limitation in the current mechanical testing of 3D tendon constructs is the absence of a suitable control group. Due to its extreme delicacy, the vehicle-treated construct proved impractical for uniaxial mechanical testing, whereas TGFβ1-treated samples exhibited sufficient strength for mechanical testing. Consequently, the conclusive determination of the TGFβ1 effect on biomechanical properties remains incomplete in this study. Another limitation of the mechanical study is that the constructs contain two layers of tissue structure. Therefore, the measured mechanical properties do not fully represent the properties of peripheral tendon-like structures. Thus, fully matured uni-structured tendon constructs need to be generated to understand the precise mechanical properties of the constructs.

Our scaffold-free 3D tendon culture can be an in vitro system to investigate the underlying cellular and molecular mechanisms regulating tendon development and organization. One limitation of our study is the small sample size for statistical analysis with a two-way ANOVA. In future studies involving this construct, we aim to enhance our statistical analysis by increasing the sample size. The future direction of this study will be inducing the cells into a further matured stage expressing higher levels of Col1a1 and Tenomodulin with increased collagen fibril diameters. Further maturation of tendon constructs could improve the mechanical properties of current 3D tendon constructs. In addition, genetic manipulation is feasible for our 3D tendon culture using an adenovirus system, which suggests the potential usage of our constructs for in vitro genetic studies for specific gene functions in tendon development and organization. We can also utilize our constructs for the screening of tenogenic factors using a small molecule library. Application of our constructs for in vitro studies can provide unique insight and understanding of biological mechanisms in tendon and bioengineered graft models.

## Data Availability

The data that support the findings of this study are available within the manuscript.
